# Methyltransferase‐like 3‐mediated N6‐methyladenosine modification of miR‐7212‐5p drives osteoblast differentiation and fracture healing

**DOI:** 10.1111/jcmm.15284

**Published:** 2020-04-19

**Authors:** Bobin Mi, Yuan Xiong, Chenchen Yan, Lang Chen, Hang Xue, Adriana C. Panayi, Liangcong Hu, Yiqiang Hu, Wu Zhou, Faqi Cao, Guohui Liu

**Affiliations:** ^1^ Department of Orthopaedics Union Hospital Tongji Medical College Huazhong University of Science and Technology Wuhan China; ^2^ Division of Plastic Surgery Brigham and Women’s Hospital Harvard Medical School Boston MA USA

**Keywords:** fracture, m6A, METTL3, miRNA

## Abstract

N6‐methyladenosine (m6A) modification has been reported in various diseases and implicated in increasing numbers of biological processes. However, previous studies have not focused on the role of m6A modification in fracture healing. Here, we demonstrated that m6A modifications are decreased during fracture healing and that methyltransferase‐like 3 (METTL3) is the main factor involved in the abnormal changes in m6A modifications. Down‐regulation of METTL3 promotes osteogenic processes both in vitro and in vivo, and this effect is recapitulated by the suppression of miR‐7212‐5p maturation. Further studies have shown that miR‐7212‐5p inhibits osteoblast differentiation in MC3T3‐E1 cells by targeting FGFR3. The present study demonstrated an important role of the METTL3/miR‐7212‐5p/FGFR3 axis and provided new insights on m6A modification in fracture healing.

## 1 INTRODUCTION

Injurious falls, traffic accidents and osteoporosis increase the risk of fractures. Although external and internal fixation yields suitable results in treating fractures, some cases of failure are inevitable. This has encouraged researchers to investigate the mechanisms underlying fracture healing, a complex biological process mediated by various factors. Previous studies have verified many pro‐ and anti‐fracture healing cytokines, small RNAs and therapeutic agents.^1^, ^2^, ^3^, ^4^

Since its first description in 1974, N6‐methyladenosine (m6A) has received extensive attention for mediating abundant internal modifications in eukaryotic messenger RNAs (mRNAs).^5^, ^6^ The presence of m6A in transcripts results in diverse cellular functions, such as pre‐mRNA splicing, nuclear transport, stability, translation and microRNA biogenesis,^7^ and is dynamically regulated by functional interplay among the catalytic proteins of methyltransferase‐like 3 (METTL3), methyltransferase‐like 14 (METTL14), Wilms tumour 1‐associated protein (WTAP), KIAA1429, fat mass and obesity‐associated protein (FTO), and alkB homology 5 (ALKBH5). The maturation process of miRNA is suppressed along with decreases in m6A levels.^8^ Inhibition of miRNA expression affects the post‐transcriptional regulation of mRNA.^9^ The diverse roles of miRNAs in fracture healing have been well documented.^10^, ^11^ This led us to investigate the m6A modification status during facture healing and whether modified m6A levels affect the miRNA maturation process, which subsequently impacts fracture healing.

Here, we assessed the role of m6A modification in fracture healing. Further, we investigated the mechanisms underlying m6A modification during fracture healing.

## 2 MATERIAL AND METHODS

### 2.1 Femoral fracture model

A total of 120 C57BL/6J mice (age, 6 weeks) were purchased from the Center of Experimental Animals, Tongji Medical College, Huazhong University of Science and Technology, China. All animal experiments were approved by the Institutional Animal Care and Use Committee of the said university. After the mice were anesthetized with 10% chloral hydrate (0.3 mL/100 g body weight), the femoral fracture model was created. Briefly, a longitudinal incision was made on the skin and the muscles were separated to expose the femur. A transverse osteotomy was performed in the mid‐diaphysis of the femur, and the bones were stabilized by inserting a 23‐gauge intramedullary needle. Equal amounts (100 μL) of phosphate‐buffered saline (PBS), plasmid METTL3 and agomiR‐7212‐5p (10 mg/kg body weight) were locally injected into the femoral fracture site. Local injection was administrated on days 0, 4 and 7. The mice were sacrificed at a designated time, and callus samples were collected for Western blot, qRT‐PCR and microCT analysis.

### 2.2 Radiographic analysis of callus formation

The entire femoral fracture site from each mouse was scanned ex vivo using the SkyScan 1176 scanner microCT system (BRUKER) and reconstructed as three‐dimensional images. Bone volume (BV), total volume (TV), BV/TV and bone mineral density (BMD) were calculated to evaluate the degree of fracture healing. Bone radiographs were taken using an in vivo FX PRO imaging system (BRUKER).

### 2.3 In vivo tracking

Cy3‐labelled miR‐7212‐5p was locally injected into the femoral fracture site of C57BL/6J mice on days 0, 4 and 7. The mice were anesthetized and observed under a bioluminescence system (BRUKE) on the above‐mentioned days, and fluorescence images for miR‐7212‐5p distribution were acquired under 740 nm excitation and 790 nm emission filters.

### 2.4 Cell culture

Mouse osteoblast precursor cells MC3T3‐E1 were cultured in α‐minimum essential medium (Hyclone) containing 10% foetal bovine serum (Gibco) and 1% penicillin and streptomycin (Hyclone). For transfection of miRNA (200 μmol/L) and siRNA (50 nmol/L), lipofectamine 3000 (Invitrogen) was used according to the manufacturer's instructions. AntagomiR‐7212‐5p, antagomiR‐NC, agomiR‐7212‐5p, agomiR‐NC and small interfering RNA (siRNA) of METTL3 and FGFR3 were synthesized by GenePharma. The plasmid METTL3 was purchased from the Public Protein/Plasmid Library (#BC001650). All cells experiments were independently repeated thrice.

### 2.5 RNA m6A quantification

Total RNA was isolated using TRIzol reagent (Invitrogen). Nanodrop (Thermo Scientific) was used to quantify the RNA concentration. The m6A content in total RNA was measured using the m6A RNA methylation quantification kit (Abcam), according to the manufacturer's instructions.

### 2.6 Western blot

Total proteins were extracted from the cells or calluses. Equal amounts of proteins (40 μL) were loaded for sodium dodecyl sulphate‐polyacrylamide gel electrophoresis, and the separate proteins were transferred onto polyvinylidene difluoride membranes. The membranes were incubated overnight with an appropriate primary antibody, followed by incubation with a peroxidase‐labelled secondary antibody for 2 hours at room temperature. The primary antibodies used were as follows: anti‐m6A (1:500; Abcam, #ab208577), anti‐METTL3 (1:500; Abcam, #ab195352), anti‐DGCR8 (1:500; Abcam), anti‐collagen I (1:500; Abcam, #ab34710), anti‐ALP (1:1,000; Abcam, #ab95462), anti‐osteocalcin (1:500; Abcam, #ab93876), anti‐Runx2 (1:500; Abcam, #ab23981), anti‐FGFR3 (1:1,000; Abcam) and anti‐GAPDH (1:10,000; #ab37168).

### 2.7 qRT‐PCR

Total RNA (including miRNA) was extracted from cells or calluses using TRIzol reagent (Invitrogen). Concentration of the extracted RNA was quantified using Nanodrop (Thermo Scientific). miRNA quantification was performed using a TaqMan miRNA assay (Applied system), the iScript Select cDNA synthesis kit (Bio‐Rad) and iQSupermix kits (Bio‐Rad). GAPDH and U6 snRNA were used as controls for standardizing the mRNA and miRNA concentrations, respectively. A real‐time PCR system with a comparative Ct method (2^−ΔΔCt^) was used to quantify RNA expression. Sequences of the primers used to probe miRNA and mRNA are shown in Table S1.

### 2.8 3ʹ‐Untranslated region (UTR) cloning and luciferase assay

Fragments of the 3ʹUTR of *FGFR3* and the miR‐26a‐5p binding site were amplified using PCR and then subcloned into pGL3 vector (Promega). Binding‐region mutations were obtained using the Quik Change Site‐Directed Mutagenesis Kit (Stratagene). For the luciferase assay, MC3T3‐E1 cells were seeded into a 96‐well plate. Then, cells were cotransfected with WT‐ or mutant‐type FGFR3 3ʹUTR‐Luc reporter plasmid and miR‐NC or miR‐7212‐5p. After 48 hours, the cells were lysed, the lysates were harvested, and luciferase activity was evaluated using the dual‐luciferase reporter assay system (Promega). The activity of luciferase activity was normalized against that of firefly luciferase.

### 2.9 RNA immunoprecipitation

For RNA immunoprecipitation, 2 × 10^7^ control cells and METTL3 knockdown cells were UV‐cross‐linked and lysed using the Magna RIP^TM^ kit (Millipore, MA, USA), according to the manufacture's instructions. The extracts were immunoprecipitated using α‐DGCR8 antibody (Abcam) or IgG as a control overnight at 4°C. Then, the immunoprecipitated protein‐RNA complexes were assessed using Western blot.

For the m6A‐pri‐miRNA and DGCR8‐pri‐miRNA binding experiments, the overexpressed METTL3 cells were UV‐cross‐linked and lysed using the Magna RIP™ kit (Millipore). The extracts were immunoprecipitated with an anti‐m6A antibody and DGCR8 antibody or IgG as a control. Beads were incubated with 200 µL of proteinase K (10 mg/mL), and RNA was extracted using phenol:chloroform:isoamyl alcohol (25:24:1). The extracted RNA was used to synthesize cDNA, which was subjected to qRT‐PCR using specific pri‐miRNA primers (normalized to input).

### 2.10 Identification of differentially expressed miRNAs

Microarray data (GSE76197) were obtained from the gene expression omnibus (GEO) database. GSE76197 is a free microarray series that evaluates the miRNAs expression profiles of mice femoral fracture callus on days 0, 3, 5, 7, 10 and 14. The down‐regulated, differentially expressed miRNAs were identified using the parameters *P* ≤ .05 and log2FC ≤ −2.

### 2.11 miRNA target site prediction

Target scan (http://www.targetscan.org) and miRDB (http://mirdb.org/) were used to predict miRNAs target genes. In addition, gene lists on PubMed were searched for fracture‐related genes.

### 2.12 Alkaline phosphatase (ALP) staining

ALP staining was assessed using the BCIP/NBT alkaline phosphatase colour development kit (Beyotime). Briefly, cells were washed with PBS and then fixed with 4% paraformaldehyde. The cells were then incubated in BCIP/NBT liquid substrate for 24 hours. The entire procedure was performed in the dark.

### 2.13 Alizarin red staining

Cells were cultured in 6‐well plates in osteogenic medium (Cyagen Biosciences) to induce osteoblast mineralization for 21 days. After that, the cells were washed with PBS and fixed with 4% paraformaldehyde. The cells were then incubated with 500 μL 0.5% alizarin red stain for 15 minutes and rinsed with ddH_2_O on an orbital shaker for 5 minutes.

### 2.14 Statistical analysis

All data have been presented as mean ± standard deviation for three independent experiments. One‐way analysis of variance with a post hoc test was performed to test between‐group differences. Student's *t* test was employed to analyse statistical differences between two groups. All statistical analyses were performed using SPSS version 22.0. *P *＜ .05 was considered statistically significant.

## 3 RESULTS

### 3.1 m6A level was suppressed during fracture healing

**FIGURE 1:**
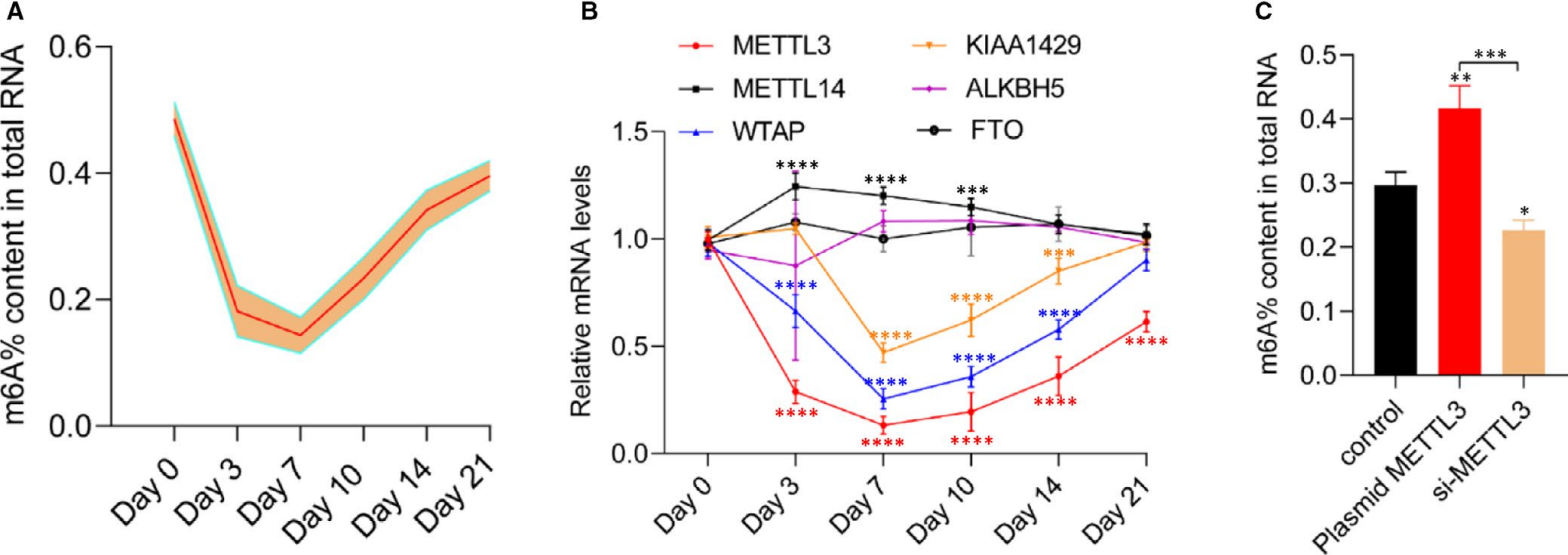
Decreased METTL3 expression contributes to lower levels of m6A methylation during fracture healing. A, Percentage of m6A in total RNAs in callus samples during fracture healing (n = 5); B, mRNA levels of m6A modification‐associated genes in calluses during fracture healing (n = 5); C, percentage of m6A in total RNAs in METTL3 overexpression or knockdown in MC3T3‐E1 cells. Data are expressed as mean ± SD. *P < 0.05, **P < 0.01, ***P < 0.001 and ****P < .0001

To elucidate the potential role of m6A modification in fracture healing, we first examined the total levels of m6A at days 0, 3, 7, 10, 14 and 21. Our data revealed that the m6A levels were noticeably decreased during the first 7 days of fracture and then gradually increased with fracture healing (Figure 1A). To identify the catalytic proteins that were mainly modified, we measured the METTL3, METTL14, WTAP, KIAA1429, FTO and ALKBH5 levels during fracture healing using qRT‐PCR. The METTL3, WTAP and KIAA1429 levels were significantly down‐regulated during the first 7 days following the fracture. In contrast, no significant changes were observed in the ALKBH5 and FTO levels. Interestingly, METTL14 levels were significantly increased during the first 10 days after fracture (Figure 1B). To verify whether METTL3 induces m6A methylases, METTL3 was overexpressed or its expression was inhibited by transfecting plasmid METTL3 or siRNA METTL3 into MC3T3‐E1 cells and measuring the m6A levels. As expected, METTL3 overexpression induced m6A levels, whereas METTL3 knockdown suppressed m6A levels in MC3T3‐E1 cells (Figure 1C). Taken together, these data suggested that down‐regulated METTL3 was the main factor determining the modified m6A expression during fracture healing.

### 3.2 Local administration of METTL3 suppressed fracture healing in vivo

**FIGURE 2:**
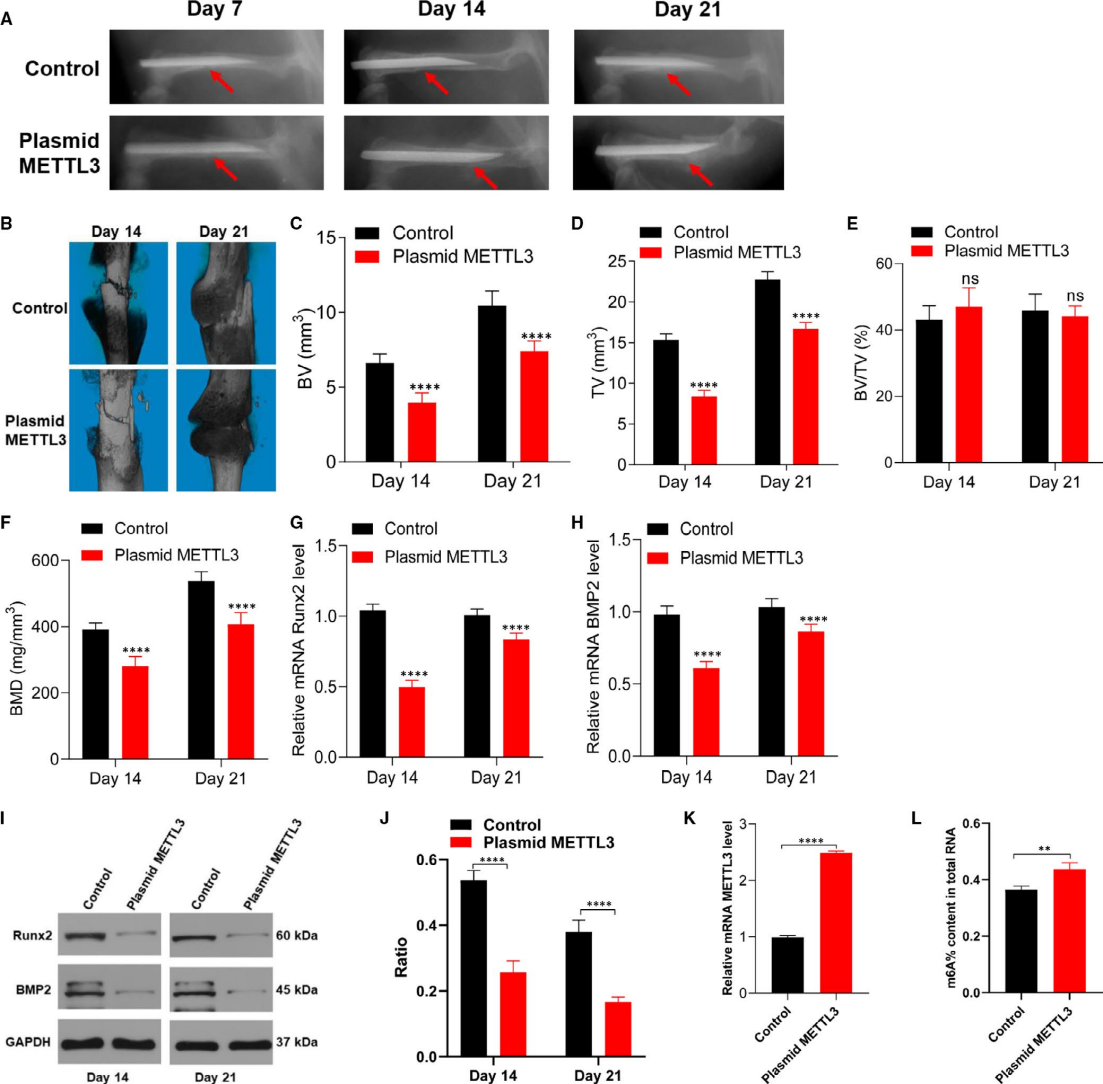
Overexpression of METTL3 delays femoral fracture healing in mice. A, Radiological changes in the femur of mice on days 7, 14 and 21 post‐fracture. B, μCT images of the fractured femur on days 14 and 21. C‐F, BV, TV, BV/TV and BMD of calluses on days 14 and 21. G‐J, Expression levels of Runx2 and BMP2 from mice calluses on days 14 and 21 were quantified using qRT‐PCR and Western blot. K, METTL3 level in the calluses on day 21 was evaluated using qRT‐PCR. L, Percentage of m6A in total RNAs in the calluses on day 21. n = 5. Data are expressed as mean ± SD. ****P < .0001

To verify the function of METTL3 in vivo, we created a femoral fracture in C57BL/6J mice. Equal volumes of PBS and plasmid METTL3 were injected locally into the fracture site on days 0, 4 and 7. Fracture healing in the mice was then evaluated on days 14 and 21. X‐ray results showed that the callus was more prominent and the fracture gaps were smaller in the control group, both on post‐fracture days 14 and 21, whereas the fracture gaps were still obvious in the plasmid METTL3 group on post‐fracture day 21 (Figure 2A). In accordance with the X‐ray results, μCT images and data showed that the total volumes and bone volumes of the calluses were significantly greater in the control group on post‐fracture days 14 and 21; in contrast, the plasmid METTL3 group exhibited lower BV, TV and BMD of the calluses (Figure 2B‐F). In addition, the qRT‐PCR and Western blot results showed that the osteogenesis‐related genes (*BMP2* and *Runx2*) exhibited significantly lower expression in the plasmid METTL3 group than in the control group (Figure 2G‐J). Furthermore, the METTL3 level was significantly increased after the mice were treated with plasmid METTL3, which also up‐regulated the content of m6A in the calluses. Thus, up‐regulation of METTL3 delays fracture healing.

### 3.3 Inhibition of METTL3 promotes osteoblast differentiation in vitro

**FIGURE 3:**
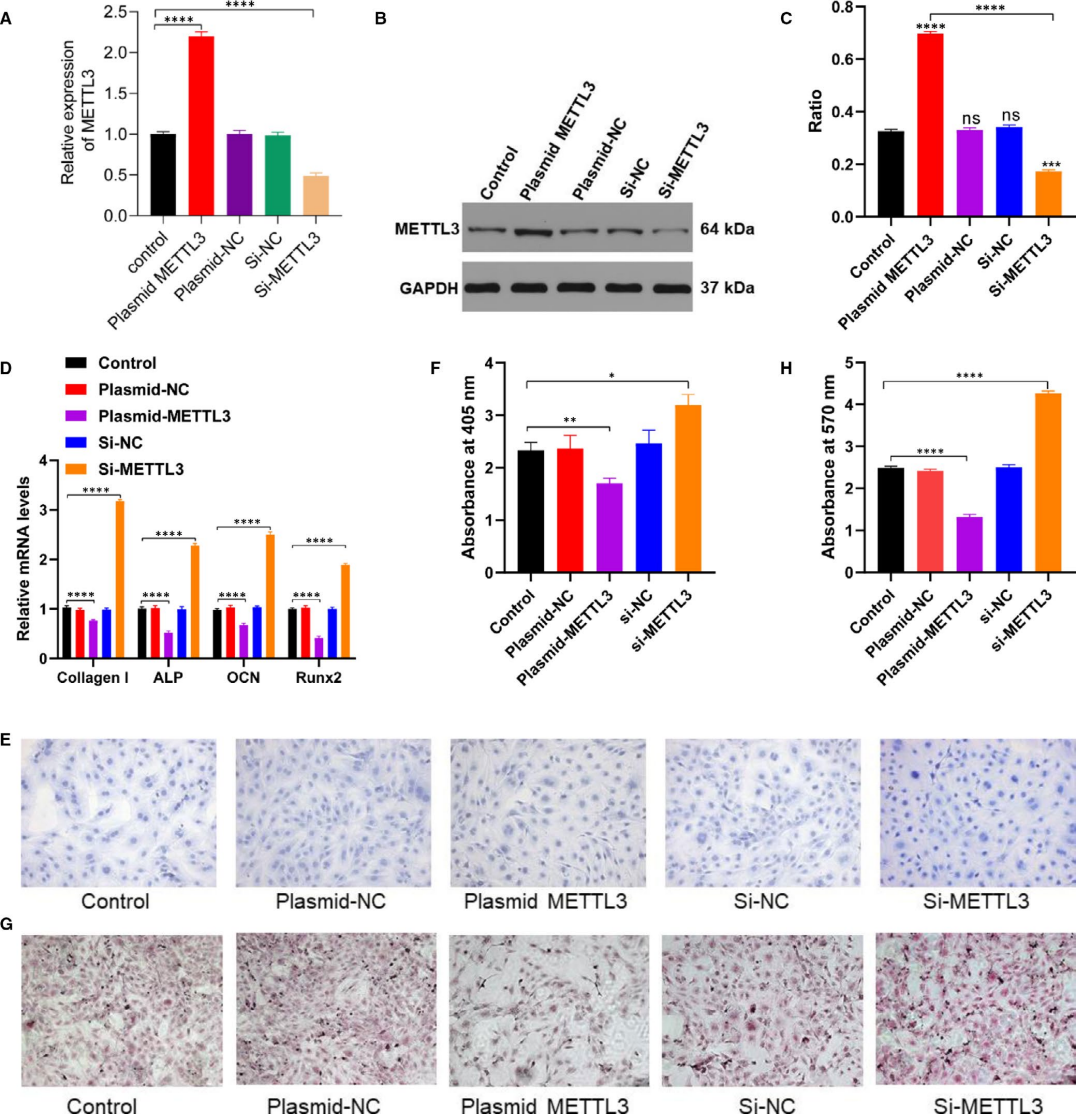
METTL3 inhibits osteoblast differentiation and matrix mineralization. A, Relative levels of METTL3 in MC3T3‐E1 cells after transfection with PBS, plasmid METTL3, plasmid‐NC, si‐NC and si‐METTL3 were detected using qRT‐PCR. B, Western blot detected the expression of METTL3 in MC3T3‐E1 cells after transfection with PBS, plasmid‐METTL3, plasmid‐NC, si‐NC and si‐METTL3. C, Quantification of the ratio of METTL3/GAPDH in (B) groups. D, mRNA level of collagen I, ALP, OCN and Runx2 was detected using qRT‐PCR. E, ALP staining of MC3T3‐E1 cells after transfection with PBS, plasmid‐NC, plasmid METTL3, si‐NC and si‐METTL3. F, Quantification of the absorbance at 405 nm in (E) groups. G, Alizarin red staining of MC3T3‐E1 cells after transfection with PBS, plasmid‐NC, plasmid METTL3, si‐NC and si‐METTL3. H, Quantification of the absorbance at 570 nm in (G) groups. Data are expressed as mean ± SD. Scale bar = 50 μm. All experiments were performed in triplicates. ****P < .0001

To further verify the effect of METTL3 in vitro, we first transfected the MC3T3‐E1 cells with PBS, plasmid METTL3, plasmid‐NC, siRNA‐NC and siRNA METTL3. Our data showed that METTL3 was significantly up‐regulated in the plasmid METTL3 group (Figure 3A‐C). METTL3 overexpression markedly decreased the level of osteogenesis‐related genes (Figure 3D). Similar results were also observed in the ALP staining and alizarin red staining (Figure 3E‐H), indicating that METTL3 acts as a negative regulator during fracture healing.

### 3.4 METTL3‐dependent m6A methylation mediates miR‐7212‐5p maturation via DGCR8

**FIGURE 4:**
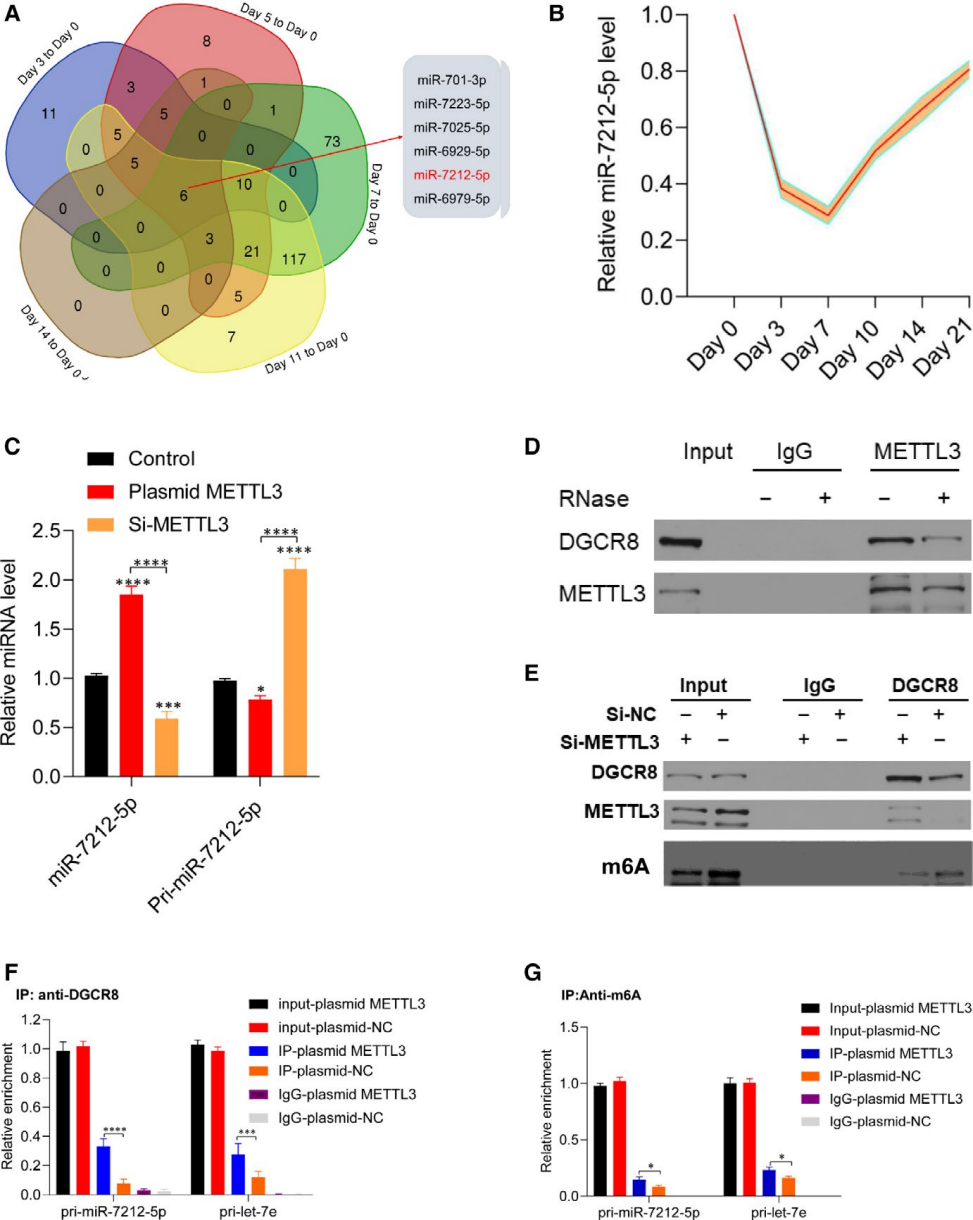
METTL3‐dependent m6A methylation mediates miR‐7212‐5p maturation via DGCR8. A, Co‐expression of down‐regulated miRNAs during fracture healing. B, Level of miR‐7212‐5p during fracture healing was detected using qRT‐PCR. N = 5. C, miR‐7212‐5p and pri‐miR‐7212‐5p levels were detected in METTL3‐overexpressing cells and METTL3‐knockdown cells using qRT‐PCR. D, Coimmunoprecipitation of the METTL3‐interacting protein DGCR8. E, Immunoprecipitation of DGCR8, METTL3, and associated RNAs from control and METTL3‐knockdown cells. (F) Immunoprecipitation of m6A‐modified RNAs in control or METTL3‐overexpressing cells to detect the levels of pri‐miR‐7212‐5PM6A modification. G, Immunoprecipitation of DGCR8‐associated RNAs in control or METTL3‐overexpressing cells to assess the pri‐miR‐7212‐5p binding with DGCR8. Pri‐let‐7e was used as a positive control. *P < .05, ***P < .001 and ****P < .0001. Day 0: intact control sample. Day 3: post‐day 3 fracture sample. Day 5: post‐day 5 fracture sample. Day 7: post‐day 7 fracture healing. Day 11: post‐day 11 fracture healing. Day 14: post‐day 14 fracture healing. All cell experiments were performed in triplicates

Previous studies have reported that METTL3 suppresses the levels of miRNAs in an m6A‐dependent pri‐miRNA‐process manner. To screen for candidate miRNAs during fracture healing, we selected microarray data concerning fracture healing from the Gene Expression Omnibus database (GSE76197). The following six miRNAs were markedly down‐regulated: miR‐701‐3p, miR‐7223‐5p, miR‐7025‐5p, miR‐6929‐5p, miR‐7212‐5p and miR‐6979‐5p (Figure 4A). Next, we measured the expression levels of these miRNAs during femoral fracture healing in mice using callus samples. The levels of these miRNAs were found to show a similar trend as that of METTL3 levels (Figure 4B), suggesting that changes in the levels of these miRNAs during fracture healing are mediated by METTL3. We then examined if these miRNAs were regulated by METTL3 in vitro. By measuring the pri‐miRNAs and miRNAs in plasmid METTL3‐ and si‐METTL3‐transfected cells, we found that miR‐7212‐5p was significantly increased in cells overexpressing METTL3 and decreased in the METTL3‐knockdown cells (Figure 4C; Figure S1).

We then tested if METTL3 promotes pri‐miRNA processing by regulating the binding of DGCR8 with pri‐miRNA. Immunoprecipitation assay showed that METTL3 coprecipitates with DGCR8 and that RNase treatment abates this interaction, indicating that the binding of DGCR8 with METTL3 is mediated by RNAs (Figure 4D). By knocking down METTL3 in the MC3T3‐E1 cells, the level of methylated RNA bound by DGCR8 is significantly decreased (Figure 4E). Further, our data showed increased binding of pri‐miR‐7212‐5p to DGCR8 in the METTL3‐overexpressing cells when DGCR8 or m6A was immunoprecipitated in the control and overexpressed METTL3 cells (Figure 4F, G). The results obtained were consistent with those of previous studies that showed that METTL3 mediated the pri‐miRNA process by recognizing DGCR8 and binding it with pri‐miRNAs.

### 3.5 miRNA‐7212‐5p inhibits fracture healing in vivo

**FIGURE 5:**
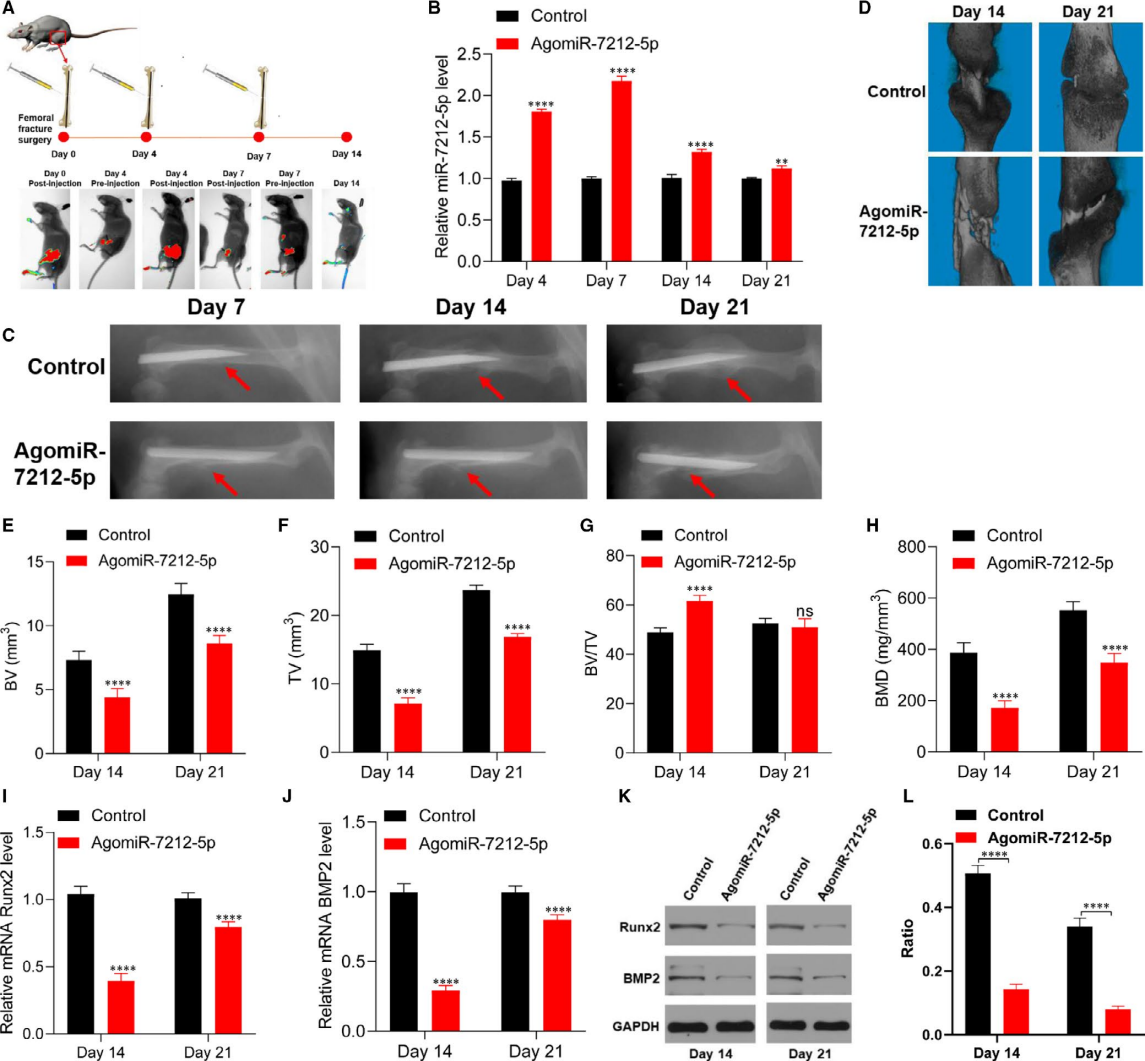
miR‐7212‐5p inhibits fracture healing in vivo. A, Fluorescent images of C57BL/6J mice before and after injection of Cy3‐labelled miR‐7212‐5p. B, Level of miR‐7212‐5p in calluses on days 4, 7, 14 and 21 after injection of PBS or agomiR‐7212‐5p. n = 5. C, X‐ray showed the fracture site of the control group and agomiR‐7212‐5p group. D, μCT images of the fractured femur on days 14 and 21. n = 5. E‐H, BV, TV, BV/TV and BMD of the calluses on post‐fracture days 14 and 21. n = 5. I‐L, Levels of Runx2 and BMP2 in mice calluses on days 14 and 21 were quantified by qRT‐PCR and Western blot. n = 5. Data are expressed as mean ± SD. **P < 0.01, ****P < .0001

To evaluate the duration of effect of miR‐7212‐5p at the fracture site, we first injected Cy3‐labelled miR‐7212‐5p into the fracture site of C57BL/6J mice and found that the signal of miR‐7212‐5p was weakened after 3 days (Figure 5A). The expression level of miR‐7212‐5p was significantly higher in the agomiR‐7212‐5p group than in the control group (Figure 5B), indicating that local injection of miR‐7212‐5p was successful. Then, we injected miR‐7212‐5p into the fracture site on days 0, 4 and 7 in the following experiments. The X‐ray results suggested that fracture gaps were observed on post‐fracture days 7 and 14 in both groups. After 21 days, the fracture gaps were still prominent in the agomiR‐7212‐5p group. However, the control group exhibited a larger callus on the fracture site (Figure 5C). In addition, the BV, TV and BMD of the callus were significantly higher in the control group than in the agomiR‐7212‐5p group, both on post‐fracture days 14 and 21 (Figure 5D‐H). The expression levels of *Runx2* and *BMP2* were also decreased in the agomiR‐7212‐5p group (Figure 5I‐L), suggesting that miR‐7212‐5p delays fracture healing.

### 3.6 miR‐7212‐5p inhibits osteoblast differentiation in vitro

**FIGURE 6:**
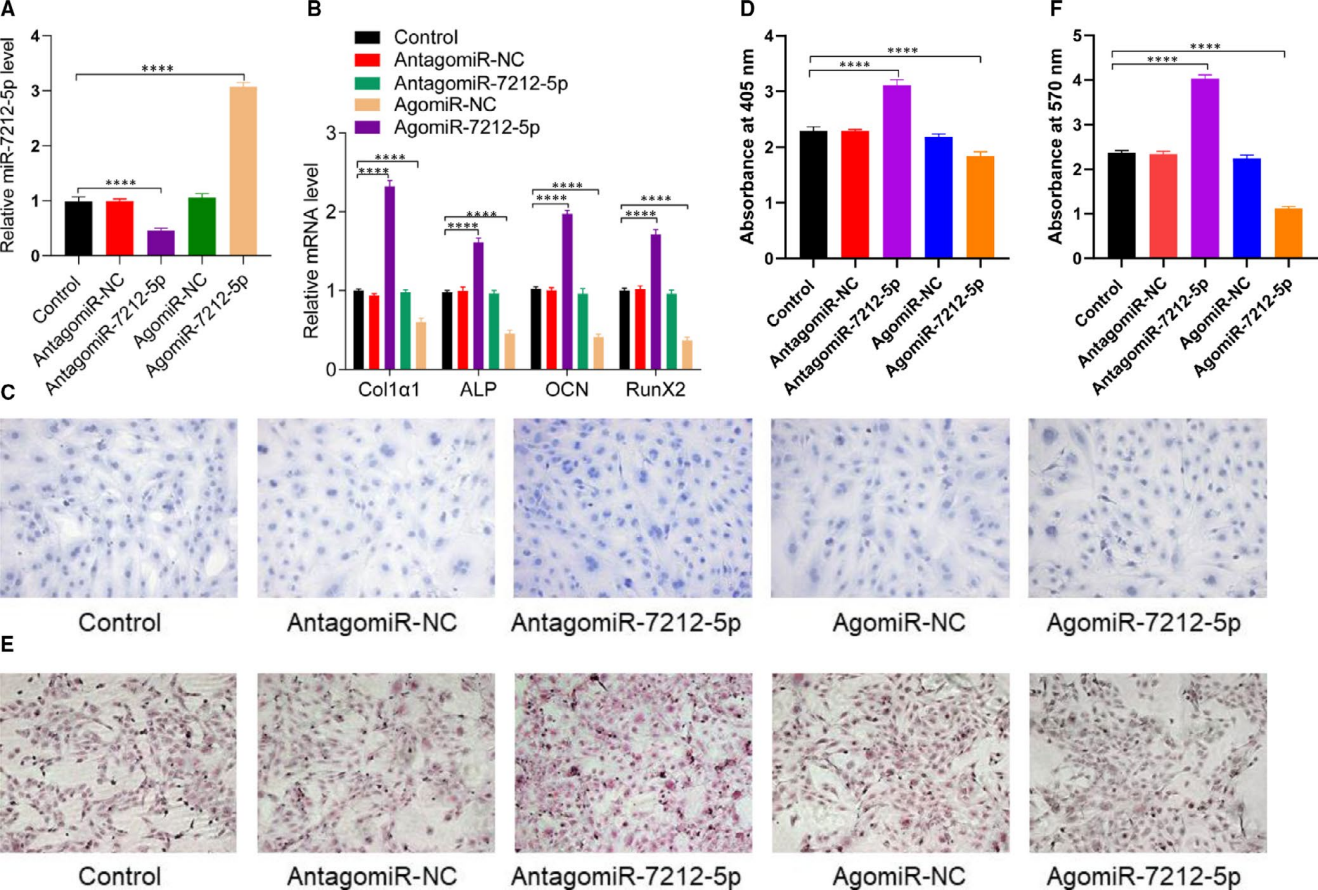
miR‐7212‐5p suppresses osteoblast differentiation. A, Relative expression of miR‐7212‐5p in MC3T3‐E1 cells after transfection with PBS, antagomiR‐NC, antagomiR‐7212‐5p, agomiR‐NC or agomiR‐7212‐5p. B, Relative levels of Collagen I, ALP, OCN and Runx2 in MC3T3‐E1 cells were quantified using qRT‐PCR. C, ALP staining of MC3T3‐E1 cells after transfection with either PBS, antagomiR‐NC, antagomiR‐7212‐5p, agomiR‐NC or agomiR‐7212‐5p. D, Quantification of the absorbance at 405 nm in (C) groups. E, Alizarin red staining of MC3T3‐E1 cells after 21 d following transfection with PBS, antagomiR‐NC, antagomiR‐7212‐5p, agomiR‐NC or agomiR‐7212‐5p. F, Quantification of the absorbance at 570 nm in (E) groups. The data are expressed as mean ± SD. Scale bar = 50 μm. All experiments were performed in triplicates. ****P < .001

To explore the role of miR‐7212‐5p during fracture healing, we transfected the MC3T3‐E1 cells with either PBS, antagomiR‐NC, antagomiR‐7212‐5p, agomiR‐NC or agomiR‐7212‐5p. Our data showed that miR‐7212‐5p was significantly increased after the cells were transfected with agomiR‐7212‐5p and decreased when transfected with antagomiR‐7212‐5p (Figure 6A). Overexpression of miR‐7212‐5p significantly suppressed osteoblast differentiation (Figure 6B) and matrix mineralization (Figure 6D‐F), indicating the adverse effects of miR‐7212‐5p on osteoblast differentiation.

### 3.7 miR‐7212‐5p mediates osteoblast differentiation by targeting FGFR3

**FIGURE 7:**
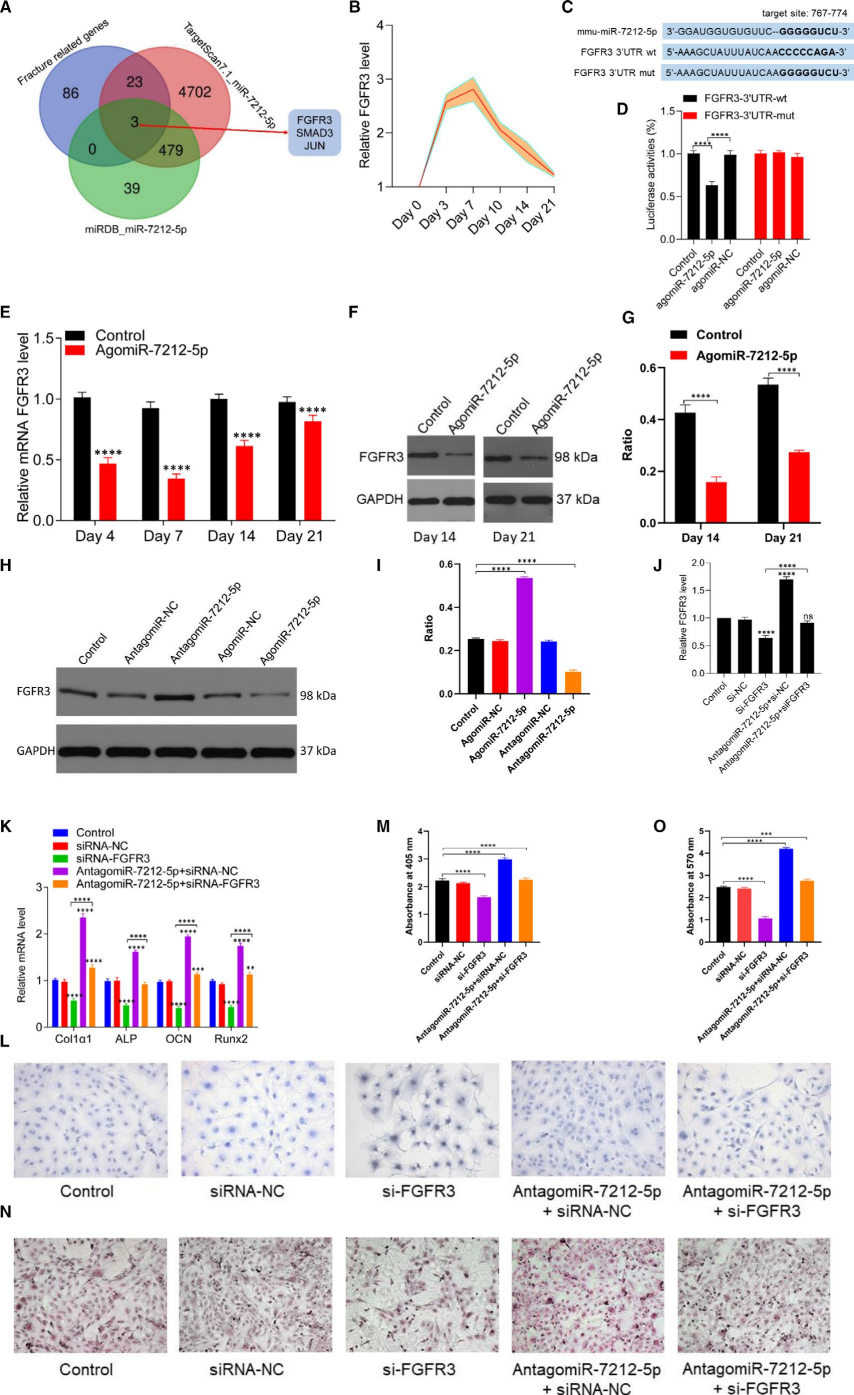
miR‐7212‐5p targets FGFR3 to inhibit osteoblast differentiation. A, Venn diagram showing that miR‐7212‐5p targets FGFR3. B, Expression level of FGFR3 during fracture healing was detected using qRT‐PCR. C, Binding site of miR‐7212‐5p with the 3ʹ‐UTR region of FGFR3. D, Luciferase reporter assay of miR‐7212‐5p with wild‐type FGFR3‐3’UTR (3’UTR‐wt) or the mutated FGFR3‐3’UTR (3’UTR‐mut). E‐G, Expression level of FGFR3 after the fracture site was injected with agomiR‐7212‐5p detected using PCR and Western blot. H and I, Western blot analysis revealed decreased FGFR3 expression after the cells were transfected with agomiR‐7212‐5p. J, qRT‐PCR was used to assess the level of FGFR3 after transfection with PBS, si‐NC, si‐FGFR3, antagomiR‐7212‐5p+si‐NC or antagomiR‐7212‐5p+si‐FGFR3. K, The levels of Col1a1, ALP, OCN and Runx2 in MC3T3‐E1 cells after they were transfected with PBS, siRNA‐NC, si‐FGFR3, antagomiR‐7212‐5p+si‐NC or antagomiR‐7212‐5p+si‐FGFR3 were quantified with qRT‐PCR. L, ALP staining of MC3T3‐E1 cells after transfection with PBS, si‐NC, si‐FGFR3, antagomiR‐7212‐5p+si‐NC and antagomiR‐7212‐5p+si‐FGFR3 for 48 h. M, Quantification of the absorbance at 405 nm in (L) groups. N, Alizarin red staining of MC3T3‐E1 cells after 21 d following transfection with PBS, si‐NC, si‐FGFR3, antagomiR‐7212‐5p+si‐NC or antagomiR‐7212‐5p+si‐FGFR3. O, Quantification of the absorbance at 570 nm in (N) groups. The data are expressed as mean ± SD. Scale bar = 50 μm. All experiments were performed in triplicates. *P < .05, **P < .01 and ***P < .001

To search for the potential target genes of miR‐7212‐5p, we compiled all the target scan‐predicted genes, miRbase‐predicted genes, and fracture‐related genes and performed Venn analysis. Our results showed that FGFR3 may be a target of miR‐7212‐5p (Figure 7A). Subsequently, we found that FGFR3 level was significantly elevated during the first 7 days following the fracture (Figure 7B). However, these results were contradictory with those for the miR‐7212‐5p levels, suggesting that FGFR3 is the main target gene of miR‐7212‐5p during fracture healing. The luciferase reporter assay showed that the luciferase activity was significantly decreased in the miR‐7212‐5p+FGFR3‐wt‐treated group, whereas there were no significant changes in the miR‐7212‐5p+FGFR‐mut‐treated group (Figure 7C, D). After agomiR‐7212‐5p was injected in vivo, the FGFR3 level was significantly reduced in the agomiR‐7212‐5p group than in the control group (Figure 7E‐G). Our in vitro study also showed that the FGFR3 level was significantly decreased in the agomiR‐7212‐5p group, whereas the level was significantly increased in the antagomiR‐7212‐5p group (Figure 7H‐J). The in vivo and in vitro studies collectively indicated that miR‐7212‐5p suppresses FGFR3 expression. By knocking down the FGFR3 in MC3T3‐E1 cells, its pro‐osteoblast differentiation effect was significantly suppressed, along with decreased matrix mineralization; however, antagomiR‐7212‐5p restored the impaired osteoblast differentiation and matrix mineralization in the si‐FGFR3‐treated cells of MC3T3‐E1 cells (Figure 7K‐O).

## 4 DISCUSSION

m6A modifications in eukaryotic RNA play vital roles in various biological processes, and dysregulation of the m6A level contributes to diseases such as tumour and malignant hematopoiesis.^12^, ^13^ However, the functions of m6A in fracture healing remain elusive. In the present study, we demonstrated that METTL3 inhibits osteoblast differentiation by targeting osteoblast‐related miR‐7212‐5p in an m6A‐dependent, pri‐miRNA‐processing manner. In addition, we found that miR‐7212‐5p suppressed osteoblast differentiation by targeting FGFR3 both in vitro and in vivo.

Establishing how m6A modifications affect fracture healing is important. Previous reports have shown that m6A modification plays important roles in the regulation of bone biology and osteoporosis.^14^ Among the m6A modulators, METTL3 is widely studied in various cells from bone, including bone marrow‐derived mesenchymal stem cells (BMSCs) and osteoclasts.^15^, ^16^ It has been reported that the correlation between METTL3 expression and osteogenic differentiation of MSCs was positive.^17^ However, our finding suggested that METTL3 inhibits osteogenic‐related genes in MC3T3‐E1 cells. We thought that the different cell lines treated by METTL3 might cause mixed results. For example, overexpression of METTL3 promotes gastric cancer progression^18^; however, down expression of METTL3 induces endometrial cancer.^19^ The dual role of METTL3 on osteogenic differentiation still needs further investigation in the future study.

Recent studies have reported that METTL3 mediates the progression of pri‐miRNA to miRNA.^20^ The fact that the increased level of miR‐7212‐5p was induced by METTL3 indicates that METTL3 promotes the maturation of osteoblast‐related miRNAs. Intriguingly, although METTL14 acts in complexes comprising METTL3, WTAP and KIAA1429, our results indicate that the level of METTL14 was inverted owing to the other three subunits. Previous studies have reported that the deletion of both METTL3 and METTL14 exerts a synergistic effect on the disruption of spermiogenesis,^21^ whereas other studies have reported that either METTL3 or METTL14 can effectively induce m6A modification.^22^, ^23^ In the present study, we found that the expression level of METTL14 significantly increased after the first 10 days of fracture. In our opinion, although METTL3 and METTL14 form a stable complex, their levels may not always change together in the same direction. Studies have reported an opposing trend in the expression of METTL3 and METTL14 in patients with liver cancer.^8^ These results suggest a complicated regulatory mechanism employed by m6A in fracture healing. Because METTL14 has also been known to regulate the progression of pri‐miRNA to miRNA,^8^ the elevated level of METTL14 may be associated with up‐regulation of the fracture‐related miRNA. In future studies, we will further explore the effects of METTL14 on fracture healing.

miRNAs play important roles in many biological processes.^14^, ^24^ The relationship between miRNAs and fracture healing has aroused great concern. Previous studies indicated that miRNAs mediate fracture healing through regulating of their target genes.^25^, ^26^ Fibroblast growth factor (FGF) signalling is reportedly involved in skeletal development.^27^ Bone regeneration is accelerated by the activation of the FGF receptor signalling pathway,^28^ whereas disruption of FGFR3 results in decreased endochondral ossification, thereby prolonging the growth period of long bones.^29^ In the present study, we demonstrated that agomiR‐7212‐5p suppresses fracture healing by inhibiting FGFR3. The effective timing for miRNA administration in mouse models for fracture healing has not been well established till date, and the methods of injection vary from study to study.^30^, ^31^ Because blood flow to the bones is not as abundant as that to the heart or liver, we injected miRNA directly into the fracture site. The results from the animal experiments showed that the miR‐7212‐5p level was gradually increased at day 7 post‐fracture. Given these results, we demonstrated that the injection of miR‐7212‐5p directly into the fracture site on post‐fracture days 0, 4 and 7 is advisable.

It is impossible to obtain calluses from patients during fracture healing process. To study the relative roles of METTL3, miR‐7212‐5p and FGFR3 during fracture healing, we harvested calluses from mice with femoral fractures. Fracture healing is a complex process, and further studies are warranted to verify the effects of m6A methylation in fracture healing.

In summary, our results indicate that METTL3 regulates miRNA maturation during fracture healing. By suppressing miR‐7212‐5p expression, METTL3 silencing promotes osteoblast differentiation both in vitro and in vivo, suggesting the crucial role of METTL3 silencing in fracture healing. Overall, these results provide new insights towards studies on fracture healing in the future.

## CONFLICT OF INTEREST

All authors declare no conflict of interest.

## AUTHORS' CONTRIBUTION

BM and GL designed the experiments. WZ and LH performed the experiments. YX, LC and CY wrote the manuscript. FC and HX collected the data and analysed the results. YH performed the X‐ray and micro CT. All authors have read and approved the final manuscript.

## Data Availability

All relevant data are available from the corresponding authors upon reasonable request.
