# Corrigendum

**DOI:** 10.1111/jcmm.15893

**Published:** 2020-12-08

In Mi et al^1^, the published article contains errors in Figure 7. The correct figures are shown below. The authors confirm all results and conclusions of this article remain unchanged.

**Figure 7:**
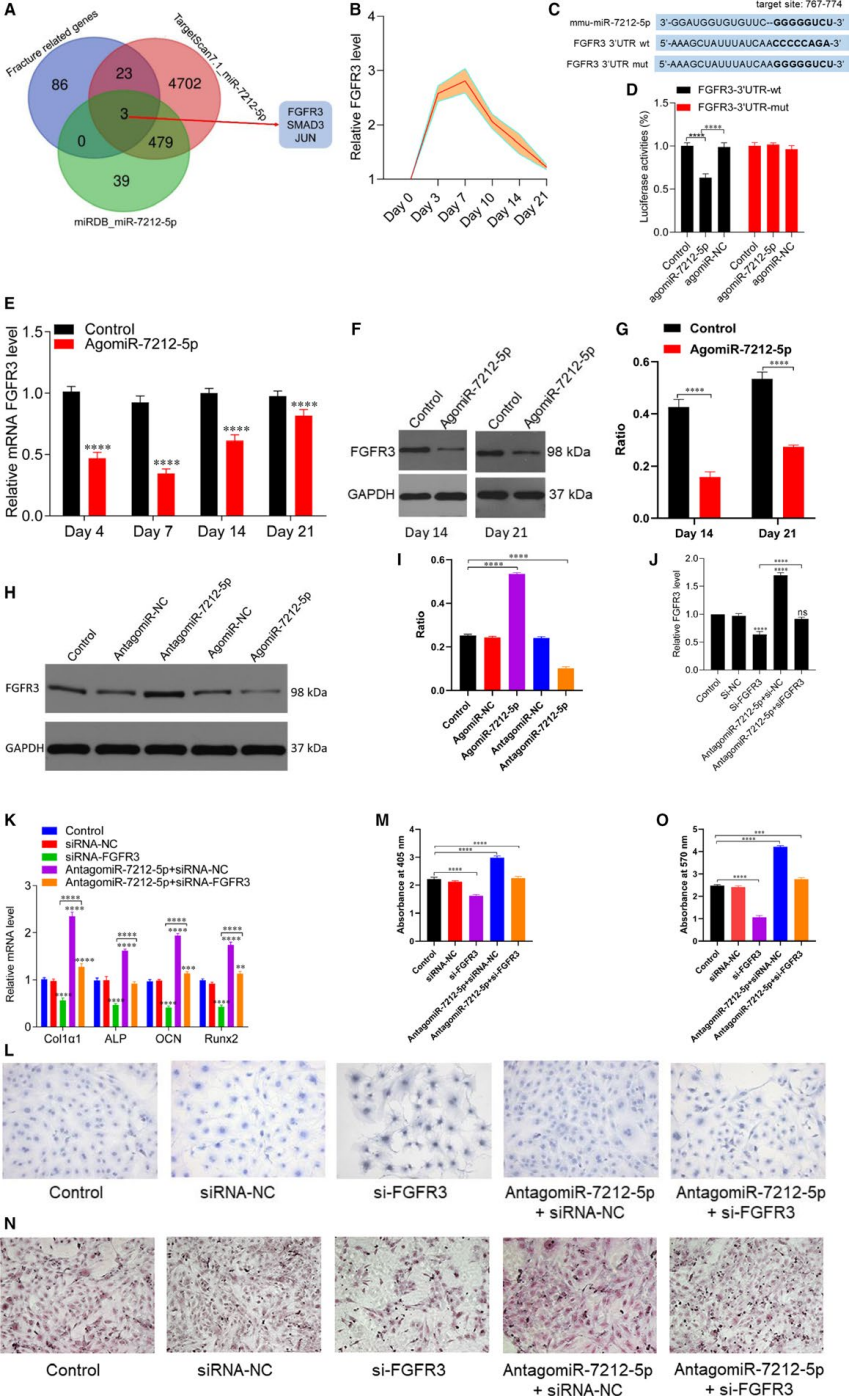
miR‐7212‐5p targets FGFR3 to inhibit osteoblast differentiation. A, Venn diagram showing that miR‐7212‐5p targets FGFR3. B, Expression level of FGFR3 during fracture healing was detected using qRT‐PCR. C, Binding site of miR‐7212‐5p with the 3ʹ‐UTR region of FGFR3. D, Luciferase reporter assay of miR‐7212‐5p with wild‐type FGFR3‐3’UTR (3’UTR‐wt) or the mutated FGFR3‐3’UTR (3’UTR‐mut). E‐G, Expression level of FGFR3 after the fracture site was injected with agomiR‐7212‐5p detected using PCR and Western blot. H and I, Western blot analysis revealed decreased FGFR3 expression after the cells were transfected with agomiR‐7212‐5p. J, qRT‐PCR was used to assess the level of FGFR3 after transfection with PBS, si‐NC, si‐FGFR3, antagomiR‐7212‐5p+si‐NC or antagomiR‐7212‐5p+si‐FGFR3. K, The levels of Col1a1, ALP, OCN and Runx2 in MC3T3‐E1 cells after they were transfected with PBS, siRNA‐NC, si‐FGFR3, antagomiR‐7212‐ 5p+si‐NC or antagomiR‐7212‐5p+si‐FGFR3 were quantified with qRT‐PCR. L, ALP staining of MC3T3‐E1 cells after transfection with PBS, si‐NC, si‐FGFR3, antagomiR‐7212‐5p+si‐NC and antagomiR‐7212‐5p+si‐FGFR3 for 48 h. M, Quantification of the absorbance at 405 nm in (L) groups. N, Alizarin red staining of MC3T3‐E1 cells after 21 d following transfection with PBS, si‐NC, si‐FGFR3, antagomiR‐7212‐5p+si‐NC or antagomiR‐7212‐5p+si‐FGFR3. O, Quantification of the absorbance at 570 nm in (N) groups. The data are expressed as mean ± SD. Scale bar = 50 μm. All experiments were performed in triplicates. *P < 0.05, **P < 0.01 and ***P < 0.001
