# Supplementary figures and images for: Methyltransferase‐like 3‐mediated N6‐methyladenosine modification of miR‐7212‐5p drives osteoblast differentiation and fracture healing
Source: J Cell Mol Med. 2020 Apr 19;24(11):6385–96. doi: 10.1111/jcmm.15284 (PMC7294157; doi:10.1111/jcmm.15284)

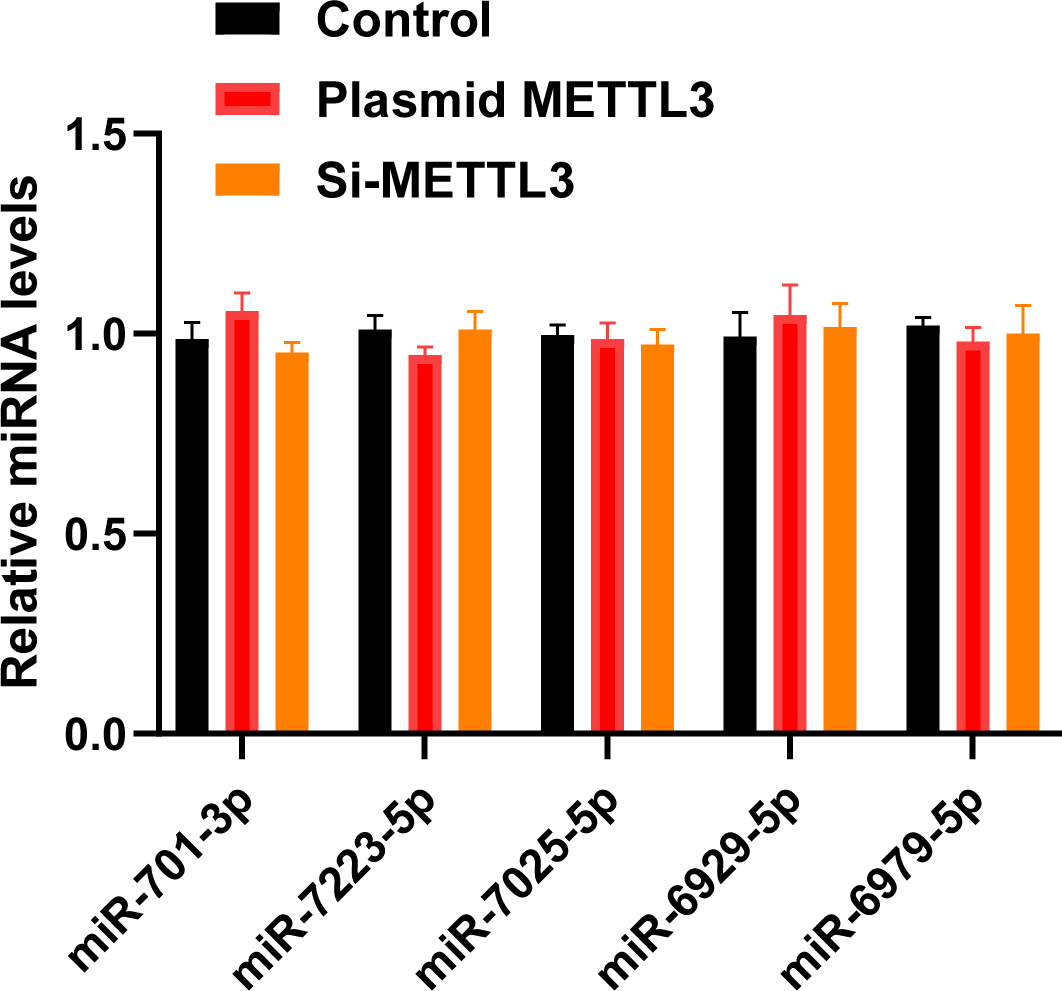

Supplement: Supplementary file 1 [file JCMM-24-6385-s001.tif]
