# Supplementary material for: Methyltransferase‐like 3‐mediated N6‐methyladenosine modification of miR‐7212‐5p drives osteoblast differentiation and fracture healing
Source: J Cell Mol Med. 2020 Apr 19;24(11):6385–96. doi: 10.1111/jcmm.15284 (PMC7294157; doi:10.1111/jcmm.15284)
Supplement: Supplementary file 2 [file JCMM-24-6385-s002.doc]

**Supplementary Table 1 miRNAs and mRNA primer sequence.**

| **microRNAs or gene name** | **Primer sequence (5’ to 3’)** |
| --- | --- |
| miR-7212-5p-Forward | ACACTCCAGCTGGGTCTGGGGGCTTGTGT |
| miR-7212-5p | TGGTGTCGTGGAGTCG |
| miR-U6-Forward | CTCGCTTCGGCAGCACA |
| miR-U6-Reverse | AACGCTTCACGAATTTGCGT |
| FGFR3-Forward | GAGGAGCTGATGGAAACTGATG |
| FGFR3-Reverse | ATTCCAAGGACACCTGTCGC |
| ALP-Forward | TGACTACCACTCGGGTGAACC |
| ALP-Reverse | TGATATGCGATGTCCTTGCAG |
| COL1α1-Forward | CTGACTGGAAGAGCGGAGAG |
| COL1α1-Reverse | CGGCTGAGTAGGGAACACAC |
| OCN-Forward | TTCTGCTCACTCTGCTGACCC |
| OCN-Reverse | CTGATAGCTCGTCACAAGCAGG |
| Runx2-Forward | CGCCACCACTCACTACCACAC |
| Runx2-Reverse | TGGATTTAATAGCGTGCTGCC |
| BMP2-Forward | ATGTGAGGATTAGCAGGTCTTTG |
| BMP2-Reverse | TCTCTTGCAGCTGGACTTGAG |
| METTL3-Forward | AACAGTCAACGAAAGAACAGCAG |
| METTL3-Reverse | ATCACAAAATTCTTGCACCTGG |
| METTL14-Forward | GAGCCCCCTCTGGAAGAATAC |
| METTL14-Reverse | CTTTCGCAAGCATACTCTCCC |
| WTAP-Forward | TGGCACGGGATGAGTTAATTC |
| WTAP-Reverse | ACTCCTGCTGTTGCTGCTTTAG |
| KIAA1429-Forward | CTGTCTGACCCTGGCAATATATG |
| KIAA1429-Reverse | TCTCACCATCAGCATGCTTTAG |
| ALKBH5-Forward | ATGAAATCACTCACTGCATACGG |
| ALKBH5-Reverse | ACAGGCGATCTGAAGCATAGC |
| FTO-Forward | GACCTTAAGAGCAGAGCAGCCT |
| FTO-Reverse | TCAGGATCTCTGCCTTCGAAG |
| GAPDH-Forward | AGAGTGTTTCCTCGTCCCG |
| GAPDH-Reverse | CCGTTGAATTTGCCGTGA |
